# The Contribution of Magnetic Nanoparticles to Ferrogel Biophysical Properties

**DOI:** 10.3390/nano9020232

**Published:** 2019-02-08

**Authors:** Felix A. Blyakhman, Emilia B. Makarova, Fedor A. Fadeyev, Daiana V. Lugovets, Alexander P. Safronov, Pavel A. Shabadrov, Tatyana F. Shklyar, Grigory Yu. Melnikov, Iñaki Orue, Galina V. Kurlyandskaya

**Affiliations:** 1 Ural State Medical University, 620028 Ekaterinburg, Russia; feliks.blyakhman@urfu.ru (F.A.B.); emilia1907@yandex.ru (E.B.M.); fdf79@mail.ru (F.A.F.); p.shabadrov@mail.ru (P.A.S.); t.f.shkliar@urfu.ru (T.F.S.); 2 Institute of Natural Sciences and Mathematics, Ural Federal University, 620002 Ekaterinburg, Russia; safronov@iep.uran.ru (A.P.S.); grisha2207@list.ru (G.Yu.M.); 3 Ural Scientific Institute of Traumatology and Orthopaedics, 620014 Ekaterinburg, Russia; 4 Center of Specialized Types of Medical Care Institute of Medical Cell Technologies, 620026 Ekaterinburg, Russia; dyana.lougovets@mail.ru; 5 Institute of Electrophysics, Ural Division RAS, 620016 Yekaterinburg, Russia; 6 Advanced Research Facilities (SGIKER), Universidad del País Vasco UPV-EHU, 48080 Bilbao, Spain; inaki.orue@ehu.eus; 7 Universidad del País Vasco UPV/EHU, Departamento de Electricidad y Electrónica and BCMaterials, 48080 Bilbao, Spain

**Keywords:** iron oxide nanoparticles, polyacrylamide gels, ferrogels, dermal fibroblasts, cellular technologies, tissue engineering, biomedical applications, magnetic biosensing

## Abstract

Iron oxide γ-Fe_2_O_3_ magnetic nanoparticles (MNPs) were fabricated by laser target evaporation technique (LTE) and their structure and magnetic properties were studied. Polyacrylamide (PAAm) gels with different cross-linking density of the polymer network and polyacrylamide-based ferrogel with embedded LTE MNPs (0.34 wt.%) were synthesized. Their adhesive and proliferative potential with respect to human dermal fibroblasts were studied. At the same value of Young modulus, the adhesive and proliferative activities of the human dermal fibroblasts on the surface of ferrogel were unexpectedly much higher in comparison with the surface of PAAm gel. Properties of PAAm-100 + γ-Fe_2_O_3_ MNPs composites were discussed with focus on creation of a new generation of drug delivery systems combined in multifunctional devices, including magnetic field assisted delivery, positioning, and biosensing. Although exact applications are still under development, the obtained results show a high potential of LTE MNPs to be applied for cellular technologies and tissue engineering. PAAm-100 ferrogel with very low concentration of γ-Fe_2_O_3_ MNPs results in significant improvement of the cells’ compatibility to the gel-based scaffold.

## 1. Introduction

The development of bioengineering constructions based on polymeric scaffolds and living cells is a promising direction for cellular technologies and tissue engineering [1,2,3,4]. Among others, the hydrogel-like scaffolds have a high potential for different applications in the area of regenerative medicine [2,3,5,6,7]. Hydrogels comprise a polymeric network swollen in water [8]. Noteworthy, the structure of synthetic hydrogels from many points of view is quite similar to the structure and properties of the biological objects that play an important role in the performance and regulation of various cellular functions [9,10]. The cellular cytoskeleton is the most striking example of a gel-like biopolymer. In addition, hydrogels belong to the category of “smart” materials, since they can significantly change their volume and shape under change of the external factors of physical or chemical nature [10,11,12,13,14].

The typical and most common hydrogels are compounds based on polyacrylamide (PAAm). The use of PAAm gels as scaffolds for the needs of cellular technologies and tissue engineering is widely discussed in the literature [15,16,17]. The advantages of PAAm gels for biomedical applications are low toxicity, cells compatibility, and high-range elasticity [18]. The Young modulus of PAAm gels can be modified from a few to several thousands of Pascal by the synthesis conditions or external impacts. This feature is of great importance, since the elastic properties of scaffolds play a key role in controlling of adhesion, proliferation, and differentiation of living cells [19,20,21,22].

Recently, the attention of tissue engineering and magnetic biosensor research has been drawn to gels filled with magnetic nanoparticles (MNPs) [23,24,25]. Electrophysical laser target evaporation (LTE) has attracted special interest in the last 10 years due to its very high production rate and very large batch size on the order of 50 g, in contrast with many chemical techniques providing much smaller batches. The size of the batch is critical parameter for nanomedicine, as materials require many tests at different stages of drug development and nanodrugs with nanoparticles are conditioned in a single batch [26]. 

Gels filled with MNPs are called ferrogels, and have an indisputable advantage over conventional gels, being sensitive to the application of the external magnetic field. This implies that with the help of a magnetic field, the control of the ferrogel’s properties and its positioning inside a living organism is possible. The possibility of ferrogel usage for cellular scaffolds has been reported in a number of studies [27,28,29]. For instance, in our early works, we demonstrated a good compatibility of cells with polyacrylamide-based ferrogels [30,31]. In particular, it was found that the adhesive activity of human dermal fibroblasts become larger with the increase of MNPs concentration in the composite, so that at the 1.0% weight fraction of MNPs in ferrogel, the cell adhesion appeared to be comparable to the cultural plastic widely used in cellular technologies. In that research, the large size of the batch provided a unique opportunity to plan various experiments based on the same batch. This kind of study is almost absent in the literature because the majority of the techniques offer only a small batch size. Here, we use the same batch of nanoparticles, as in our previous works [30,31], in order to insure the validity of the comparison of the results.

It was suggested that the effect of iron oxide MNPs concentration on the degree of cell adhesion to ferrogels is closely correlated with the impact of particles on the elastic properties of the magnetic composite. Indeed, a gradual increase of the MNPs concentration resulted in an unexpectedly large increase in the Young modulus of ferrogels. Moreover, the relationship between the Young modulus and the adhesion index was established. At the same time, we could not completely exclude the direct effect of the MNPs on the cells’ adhesiveness to the ferrogel.

The present study addresses a role of iron oxide MNPs contributions in determining the adhesive and proliferative potential of cells. The series of PAAm gels with different polymer network cross-linking density and elastic properties, and the polyacrylamide-based ferrogel with embedded iron oxide MNPs were synthesized. We show that for the same value of Young modulus, the adhesive and proliferative activities of the human dermal fibroblasts cultivated onto the surface of the ferrogel are unexpectedly much higher than those cultivated onto the surface of PAAm gel.

## 2. Materials and Methods 

### 2.1. Nanoparticle, Gel, and Ferrogel Synthesis and Primary Characterization

**Figure 1:**
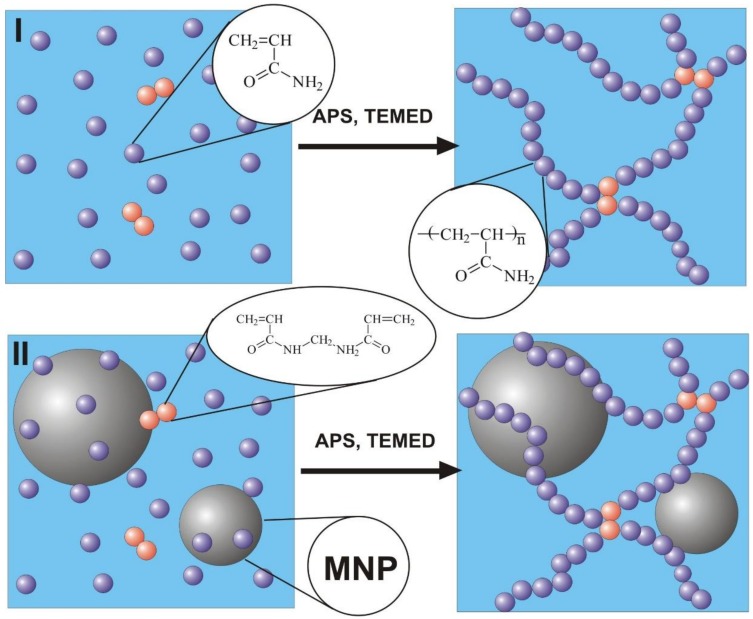
Schematic presentation of gel preparation (**I**) and ferrogel preparation (**II**).

Synthesis of polyacrylamide hydrogels was performed by free radical polymerization in 1.6 M water solution of monomer–acrylamide (AAm, AppliChem, Darmstadt, Germany) using N,N′-methylene bisacrylamide (MBAA, Merck Schuchardt, Hohenbrunn, Germany) as a cross-linking agent. The variation of networking density of hydrogels was provided by different MBAA/AAm ratios, which were set to 1:50, 1:100, 1:200, and 1:300. The series of hydrogels with increasing networking density are further denoted as PAAm-50, PAAm-100, PAAm-200, and PAAm-300. Ammonium persulfate (APS) was used as an initiator for polymerization and N,N,N′,N′-tetraethylmethylenediamine (TEMED, Merck, Schuchardt, Hohenbrunn, Germany) was used as a catalyst. The concentration of APS was 3 mM, and the concentration of TEMED was 5 mM in the reaction mixture. A schematic representation of gel preparation is given in Figure 1.

The same route was used for the synthesis of ferrogel, based on polyacrylamide with embedded γ-Fe_2_O_3_ maghemite [32] MNPs. In the case of ferrogel, however, the reaction mixture was prepared using water suspension of maghemite MNPs stabilized by 5 mM sodium citrate. Maghemite MNPs were synthesized by laser target evaporation (LTE), for which schematic presentation and detailed description were given earlier [33]. The main advantage of this technique is the large size of the individual batch. MNPs fabricated by physical condensation in the vapor have a strong tendency to aggregate [34]. By careful adjustment of LTE parameters, it is possible to avoid coalescence of the liquid droplets of the material in the vapor and prevent formation of large agglomerates.

**Figure 2:**
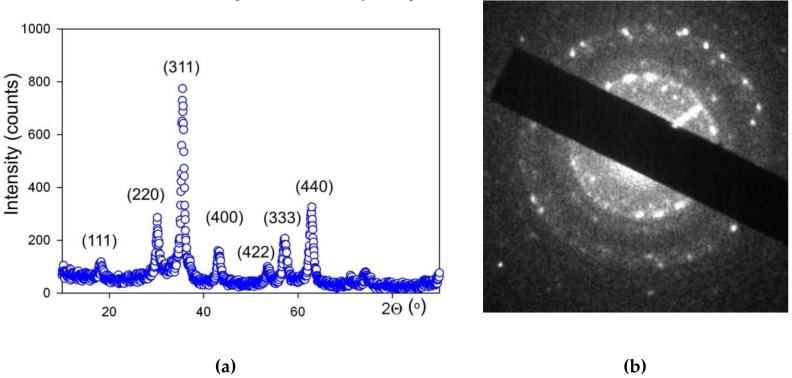
XRD pattern (**a**) and microdiffraction TEM image (**b**) of iron oxide magnetic nanoparticles (MNPs).

The X-ray diffraction characterization (XRD) was performed by operating at 40 kV and 40 mA DISCOVER D8 diffractometer (Bruker, Billerica, MA, USA) with Cu-K*α* radiation (wave length *λ* = 1.5418 Å), a graphite monochromator and a scintillation detector. The air-dried MNPs were mounted on silicon zero background plate placed in a generic sample holder. A fixed divergence and antiscattering slit were used. Bruker software TOPAS-3 with Rietveld full-profile refinement was employed for the quantitative diffractogram analysis. Transmission electron microscopy (TEM) studies of MNPs were performed using a JEOL JEM2100 microscope (JEOL Ltd., Tokyo, Japan) operating at 200 kV. The chemical composition of LTE MNPs was determined by the combination of Red-Ox titration (Schott Titroline, SCHOTT Instruments GmbH, Mainz, Germany) and the analysis of the lattice period provided by XRD and TEM (Figure 2).

The suspension of air-dried MNPs in 5 mM sodium citrate, which was used for the synthesis of ferrogel, was deaggregated by ultrasound treatment for 30 min using Cole-Parmer CPX-750 processor (Cole-Parmer Instruments Corp., Vernon Hills, IL, USA) operated at 250 W. Permanent cooling of the suspension was provided. The remained aggregates were eliminated by centrifuging at 8000 rpm for 5 min. The deaggregation of MNPs in suspension was monitored by the measurement of the hydrodynamic diameter during the preparation of the stock suspension of MNPs. The hydrodynamic diameter of MNPs by DLS using Brookhaven Zeta Plus analyzer (Brookhaven Instruments Corp., Holtville, NY, USA) in multi-size distribution mode was characterized by a fraction with median 36 nm (87.5% by weight) and a fraction with median 78 nm (12.5% by weight). The first fraction was related to individual MNPs as its median was close to the intensity averaged diameter of MNPs (31 nm) calculated using lognormal PSD obtained by graphical analysis of TEM images. The final concentration of MNPs in stock ferrofluid was 5% by weight (determined by dry solid fraction weighting with correction to the content of citrate). The stock ferrofluid was then diluted with 5 mM sodium citrate to be used in the reaction mixture for ferrogel preparation. The concentration of MNPs in ferrogel was 0.34% by weight. 

In both the case of hydrogels and ferrogels, the reaction mixture was placed between polished glass sheets 60 mm × 90 mm separated by 0.8 mm spacers, and sealed by a silicon sealer. Polymerization was performed at 25 °C for 60 min. After that, the mold was disassembled and the sheet of PAAm hydrogel was extensively washed in distilled water for 7 days with daily water renewal in order to achieve equilibrium swelling. Then hydrogel sheets were stored in Hanks’ Balanced Salt Solution (HBSS) with phenol red pH = 6.8−7.2 (PanEco Ltd, Moscow, Russia) and gentamicin (100 µg/mL) with daily renewal. Then hydrogel sheets were stored in 199 solution pH= 7.0 − 7.4, osmolarity 300 ± 20 mosmol/kg, buffering capacity ≤ 1.5 mL (PanEco Ltd, Moscow, Russia) with gentamicin (100 µg/mL).

The thickness of prepared PAAm sheets was approximately 1 mm. PAAm scaffolds in the shape of discs 13 mm in diameter were cut off the sheets to fill the wells of the standard 24-well polystyrene plate for cell culturing. The scaffolds were sterilized in autoclave at 121 °C for 20 min.

The values of water uptake by hydrogels per unit mass of dry polymer (swelling degree) were measured by gravimetry. A gel sample swollen to equilibrium was weighed and then dried to a constant weight in a thermostat at 70 °C. The weight loss related to the mass of the dry residue gave the values of swelling degree (*α*).

(1) $$\alpha =\frac{m-m_{0}}{m_{0}},$$

where *m* is the mass of a swollen gel specimen, *m_0_* is the mass of the dry residue.

For simple evaluation of the surface features of the gel and ferrogel scaffolds, 4 mm × 4 mm squares were cut off the sheets without surface damage. They were dried in the exicator. For their surface feature evaluation, we applied the same methodology which was previously used for polymer composites filled with metallic MNPs [35]. As the main problem of the polymer composite structural evaluation by electron microscopy is the charging of its surface, a thin carbon layer of about 20 nm was deposited onto the dried composite surface. Scanning electron microscopy (SEM) was performed with JEOL JSM-640 (20 kV accelerating voltage) microscope (JEOL Ltd., Tokyo, Japan) equipped with energy dispersive X-ray (EDX) fluorescent detector for elemental analysis. 

### 2.2. Gel Mechanical Properties Testing

The laboratory setup for mechanical testing of gels was used as described early [30,31]. A cylindrical ferrogel sample (~7 mm in length and ~10 mm in diameter) placed in a bath filled with Hanks’ solution was clamped vertically between two parallel plates connected to the levers of the force transducer and the linear motor equipped with an optical transducer for the gel length measurement. The measurement in a quasi-static mode was performed by a step-wise application of compressive deformation (*ε*) to a sample with force equilibration for at least 10 s at each step. The deformation step was 1–2% of the sample height and the total deformation of the sample was up to 16%. Gel tension (*σ*) was calculated as the recorded force normalized by the gel cross-sectional area. The diameter of cylindrical gel sample was monitored at every step by means of digital camera to correct the cross-section area in the course of compressive deformation. Young modulus (*E*) was determined as the slope of linear part of the curve in coordinates *ε* vs. *σ*: *tgα = σ/ε = E*. The compression test was applied for all kinds of gels (four samples for each).

### 2.3. Magnetic Properties Testing

Magnetic measurements were performed in the temperature range 5 to 300 K by a vibrating sample magnetometer (Cryogenics Ltd. VSM, London, UK) or a superconducting quantum device (Quantum Design MPMS-7, Quantum Design Inc., San Diego, CA, USA). Here we describe the high field (in a field of 6.7 × 10^3^ kA/m) value of magnetization as the saturation magnetization (Ms). Zero-field cooled (ZFC) and field cooled (FC) thermomagnetic curves were also measured for the value of applied magnetic field H = 3.95 kA/m. In order to obtain zero-field cooled data, MNPs were cooled in zero field from temperature of 300 K down to 5 K, and then the magnetization (M) was measured with an increase of the temperature under the applied field of H = 3.95 kA/m. For field cooled case, the same magnetic field H = 3.95 kA/m was applied for cooling and heating of the MNPs sample. 

### 2.4. Testing Cells’ Adhesive and Proliferative Activities 

To minimize the differentiation of cells during in vitro cultivation, the primary donor’s fibroblasts after minimal passages were used. To obtain a primary culture of dermal fibroblasts, a 0.5 cm × 1 cm skin fragment was removed during a planned surgery from a patient who had previously given written consent to pass through this procedure. The Ethics Committee of the Institute of Medical Cell Technologies (Ekaterinburg, RF) approved the study. The skin biopsy sample was dissociated using Clostridium histolyticum collagenase I (Sigma-Aldrich, St. Louis, MO, USA) with an activity of 300 U/mL for 150 min at 37 °C, after which the enzyme was inactivated, the cells were sedimented by centrifugation and resuspended in growth medium. For cultivation, a growth medium was used consisting of a mixture of Dulbecco’s Modified Eagle’s Medium (DMEM) with high glucose content and F-12 media (1:1 ratio) (Gibco, Waltham, MA, USA) with the addition of 12% fetal calf serum (Gibco, Waltham, MA, USA), glutamine (0.03%) and gentamicin (50 μg/mL of medium). Cells were grown in plastic culture flasks (Nunc, Roskilde, Denmark) in a CO_2_ incubator MCO-15AC (Sanyo/Panasonic) (37 °C, in an atmosphere of 5% CO_2_ at 100% relative humidity).

Cells were passaged when the monolayer reached 80–90% confluence. Trypsin 0.25% solution with ethylenediaminetetraacetic acid(EDTA, Gibco, Waltham, MA, USA) was used to detach cells from plastic. The number of viable cells was counted after staining them with trypan blue using a TC-20 cell counter (Bio-rad, Hercules, CA, USA). Fibroblasts of passage 8 were used in the experiment.

The sterile gel disks (diameter 13 mm, width ~1 mm) were placed into the wells of sterile 24-well plates (Techno Plastic Products, Trasadingen, Switzerland). Fibroblast suspension in growth medium was dispensed into plate wells with a seeding density of 3000 cells/cm^2^. Plates with cells were incubated in a CO_2_ incubator. Accounting for adhesive activity of fibroblasts was carried out after 12 h of incubation, and assessing the rate of cells proliferation after 4 days. All samples in the experiments were duplicated 6 times.

At the end of the incubation period, the cells on the gel disks were washed with phosphate buffer solution, after which they were fixed with 2.5% glutaraldehyde solution at 4 °C for 24 h, then dehydration was performed, by successive incubation in ethanol with increasing concentration (30%, 50%, 70%, and 95%) for 10 min corresponding to each concentration. The cytoplasm of the fixed cells was stained with a 0.3% pyrazolone yellow solution, after which the cells were washed with a buffer solution and stained with 4′,6-diamidino-2-phenylindole (DAPI, Sigma-Aldrich, St. Louis, MO, USA) according to the manufacturer’s instructions.

Cells were imaged by fluorescent microscopy at 100-fold magnification using an Axio Lab. A1 FL (Carl Zeiss, Oberkochen, Germany) fluorescence microscope in FITC channels (for pyrazolone yellow) and DAPI. For each sample, images were taken in 5 fields of view. The area of the field of view was 0.93 mm^2^. Using the software package ImageJ (Wayne Rasband, NIH), the number of cells was calculated by the number of nuclei related to the images taken, and the area and perimeter of the cells were also determined.

The data were processed using the method of descriptive statistics and they were presented in the form M´ ± m, where M´ is the arithmetic average and m is the standard error of the arithmetic average. When comparing the quantitative traits of two independent groups, the non-parametric Mann–Whitney test was used, the statistical hypothesis was considered confirmed at a significance level of *p* ≤ 0.05. Statistical data processing was performed using the application software package “STATISTICA 6.0”.

## 3. Results

**Figure 3:**
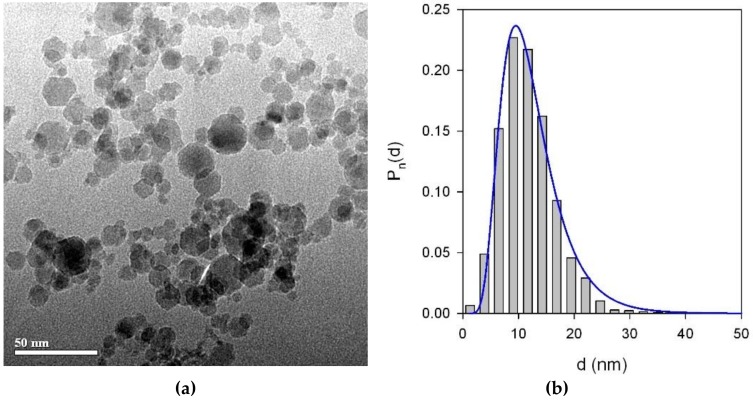
TEM micrograph (JEOL JEM2100) of maghemite γ-Fe_2_O_3_ LTE MNPs (**a**) used for the synthesis of ferrogel. Particle size distribution (**b**) was obtained by a graphical analysis of 2160 TEM images of MNPs. The line in the PSD corresponds to lognormal fitting of the experimental data.

Figure 3 shows the TEM images of MNPs with a histogram of particle size distribution (PSD). One can see that the shape of LTE MNPs is close to spherical. The observable deviation from spherical sphere to hexagon corners stems from the formation of crystalline planes on the surface of monocrystalline MNPs. One can appreciate the very close to spherical shape of LTE MNPs. PSD obtained on the basis of the TEM images analysis fits well to a lognormal distribution function with a median value of 11.7 nm and a standard deviation for the natural logarithm of the diameter = 0.423 (unitless). 

XRD analysis of MNPs showed that the crystalline structure of iron oxide MNPs corresponded to the inverse spinel lattice, space group Fd-3m. Actually, this structure refers both to magnetite (Fe_3_O_4_) and maghemite (γ-Fe_2_O_3_), which differ in the number of Fe atoms in the elementary cell [33] and in the period of the lattice. For synthesized LTE NMPs, the period was 0.8357(5) nm, maghemite was 0.8346, and magnetite was 0.8396. Red/Ox titration of MNPs gave the stoichiometric formulation of MNPs Fe_2.67_O_4_. Based on these results, we may conclude that elaborated MNPs are very close to γ-Fe_2_O_3_ More detailed analysis of the specific features of the chemical composition of LTE iron oxide MNPs is given in earlier work [33]. 

**Figure 4:**
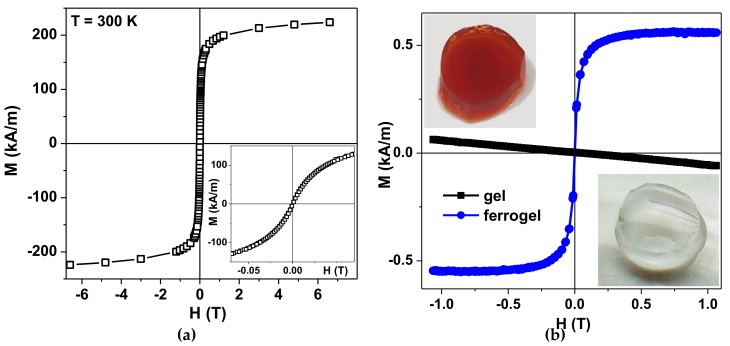
(**a**) Magnetic hysteresis loop of air-dried maghemite LTE MNPs. Inset—enlarged view for the low-field range behavior. (**b**) Magnetic hysteresis loop of gel (transparent) and ferrogel filled with maghemite LTE MNPs (opaque). MDAA/AAm ratio was 1:100, MNPs concentration in ferrogel was 0.34 wt.%.

Figure 4a shows magnetic hysteresis loop of the air-dried maghemite MNPs measured by MPMS. The inset shows the magnetization behavior in low magnetic fields. The saturation magnetization value Ms ≈ 250 kA/m is reasonable for γ-Fe_2_O_3_ MNPs of about 12 nm in diameter [32,33]. In order to understand the magnetic behavior of LTE MNPs, the core–shell model (with no inter particle coupling included) was previously proposed and tested. Each nanoparticle grain was supposed to consist of two parts: a ferrimagnetic maghemite core and a surface shell of thickness delta, in which the spins are frozen but there is no long-range magnetic order, yielding a “spin glass”-like state [33]. The complexity of the magnetic response of such a material was attributed to the large random exchange fields and the concomitant anisotropy, corresponding to the core because of the coupling to the spins of the surface. The magnetization behavior when approaching saturation and the coercivity at room temperature are consistent with the “core–shell” model proposed earlier for maghemite MNPs of similar size.

Figure 4b shows a general view of the transparent gel without MNPs and the ferrogel with MNPs together with quasistatic magnetic hysteresis loops of gel and ferrogel. Ferrogel was filled with maghemite LTE MNPs (MDAA/AAm ratio was 1:100, MNPs concentration in ferrogel was 0.34 wt.%). As expected, one can see clear diamagnetic response of the gel and an “S” shaped response of the ferrogel. The first one is due to the high water content in the gel. The second one is due to high contribution of the magnetic responses of LTE MNPs having a core–shell structure consisting of a ferrimagnetic maghemite core and a surface shell in which the spins are frozen, like in a “spin glass” [33]. Subtraction of the MNPs signal confirmed that the indicated concentration of the MNPs was defined correctly.

The values of swelling degree (*α*) in water and Medium 199 solution are given in Table 1. It is known that Medium 199, when properly supplemented, has broad species applicability for cultivation of non-transformed cells for in vitro cultivation [36]. The swelling degree of the hydrogel is the basic characteristic feature of the density of its networking. The average number of monomer units in linear subchains between cross-links of a gel network can be evaluated using the Flory–Rehner equation [37,38] based on the swelling degree equilibrium value of a gel network:

(2) $$N_{C}=\frac{V_{1}(0.5\alpha_{0}\alpha^{-1}-\alpha_{0}^{1/3}\alpha^{-1/3})}{V_{2}(\ln(1-\alpha^{-1})+\alpha^{-1}+\chi \alpha^{-2})},$$

where N_c_ is the number of monomer units among cross-links. *V_1_*, *V_2_* are molar volumes of a solvent and of a polymer, respectively, *χ* is Flory–Huggins parameter for a polymer–solvent mixture, α_0_ is the swelling degree of PAAm gel as provided by the composition of the reaction mixture in the synthesis. We used *V_1_* = 18 cm^3^/mol (water), *V_2_* = 56.2 cm^3^/mol (polyacrylamide) and *χ* = 0.12. The last two values were calculated by means of quantum mechanics molecular modeling software package CAChe7.5. The values of N_c_ for PAAm hydrogels are given in Table 1. They are increasing from PAAm-50 to PAAm-300.

The equilibrium conformation of electrically neutral polyacrylamide subchain in water is a random Gaussian coil with hindered rotation. Its mean square end-to-end distance <R^2^>, which corresponds to the distance between adjacent cross-links, can be calculated according to the equation [39]:

(3) $$\langle R^{2}\rangle =Na^{2}\frac{1-cos𝜗}{1+cos𝜗},$$

where *N* is the number of bonds in the polymeric chain, *a* is the bond length, *𝜗* is the bond angle.

We took *a* = 0.154 nm for the ordinary C–C bond, *θ =* 109.5^0^ for the bond angle, and *N* = 2*N_C_* for the number of bonds (it is two-fold larger than *N_C_* as it includes the bonds in monomer units and bonds between them). 

If considering the network of flexible pθolymeric chains, the value of the square end-to-end distance relates to the conformation of the subchains in the network, which are affixed to the cross-links by their both ends but can freely bend between cross-links. Thus, the square root of <R^2^> gives the average distance between the cross-links (L) of the polymeric network (L = <R^2^>^1/2^).

The values of the average distance between adjacent cross-links in the polymeric network of PAAm hydrogels are given in Table 1. They are compared to the values of the average geometric length (L_0_) of the subchains. The latter value, in other words, is the average length of the fully-stretched subchains. 

Both the average distance between cross-links and the average geometric length of a subchain (L_0_) steadily increase from PAAm-50 to PAAm-300. It means that the network of PAAm-50 is the densest in these series and the network of PAAm-300 is the loosest. 

Let us consider the L_0_/L ratio, which gives the ability for the subchain to uncoil and to stretch under external force. It is noticeable that the L_0_/L ratio substantially increases in this series. It means that subchains in PAAm-50 hydrogel are less coiled than subchains in PAAm-300. Less coiled subchains in the network of PAAm-50 hydrogel are more resistant to external force than more coiled subchains of PAAm-300 network. Thus, the steady increase in the L_0_/L ratio is the basic reason for the diminishing of the Young modulus of hydrogels in PAAm series. For the rigorous consideration of the influence of the polymeric network cross-link density on the Young modulus see for example [40].

**Figure 5:**
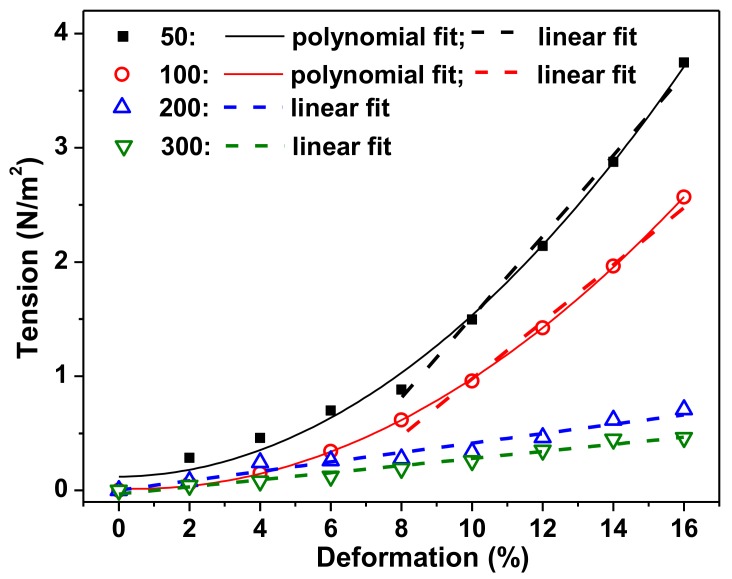
Typical examples of deformation–tension dependencies obtained in PAAm gels with different cross-linking densities of the polymer network. The equations of linear approximation for: 50: *σ* = 0.35*ε* − 2.02; 100: *σ* = 0.21*ε* − 1.09; 200: *σ* = 0.05*ε* − 0.05; 300: *σ* = 0.03*ε* − 0.06.

Figure 5 shows the typical examples of deformation–stress (*ε*–*σ*) dependences for PAAm gels with crosslinking degrees 1:50, 1:100, 1:200, and 1:300, obtained in the course of stepped compression test of the samples.

It can be seen that the steepness of the bond increases with increasing density of hydrogel crosslinking. PAAm-50 gel most clearly reacts to compression deformation, and the “*ε–σ*” bond has the greatest steepness. The graph shows the linear part of the dependencies, and the equations of linear approximation are given in the caption of Figure 5. The coefficient at the first term of the equation is equal to the tangent of the slope of the curve and corresponds to the value of the Young modulus. This corresponds to 35, 21, 5, and 3 kPa for PAAm gels with crosslinking degrees 1:50, 1:100, 1:200, and 1:300, respectively. Table 2 presents the mean values of stiffness obtained at different deformations for all kinds of tested gels.

The results of the mechanical testing of gels are in qualitative agreement with theoretical predictions based on an estimate of the degree of swelling of gels with different crosslinking densities. Indeed, the greater the stiffness of the gel, the more densely the polymer is crosslinked. Table 3 shows the average values of the Young modulus for all tested PAAm gels, including a PAAm-100 ferrogel with iron oxide MNPs in concentration 0.34 wt.%. It can be seen that the density of the PAA-50 gel of the Young modulus is an order of magnitude greater than that of the lightly cross-linked PAAm-300, but approximately the same as the ferrogel. It is important to note that the rigidity of the samples is in the body’s soft tissue range: 0.1–1 kPa for the brain, 8–17 kPa for striated muscles, 25–40 kPa for osteoid tissue [41].

The increase in the Young modulus of a gel resulting from the addition of a low concentration of MNPs is a well-established fact [30,31,42]. It reflects the direct effect of LTE MNPs on the gel elasticity. In certain cases, embedded MNPs can act as cross-linking agents of a gel network. However, it results in the diminishing of the swelling degree of the gel due to the increase in the density of its network. We did not observe a decrease of the swelling degree of ferrogel compared to the gel with the same networking density. Therefore, we assume that additional cross-linking by MNPs is negligible in the case of PAAm ferrogel.

**Figure 6:**
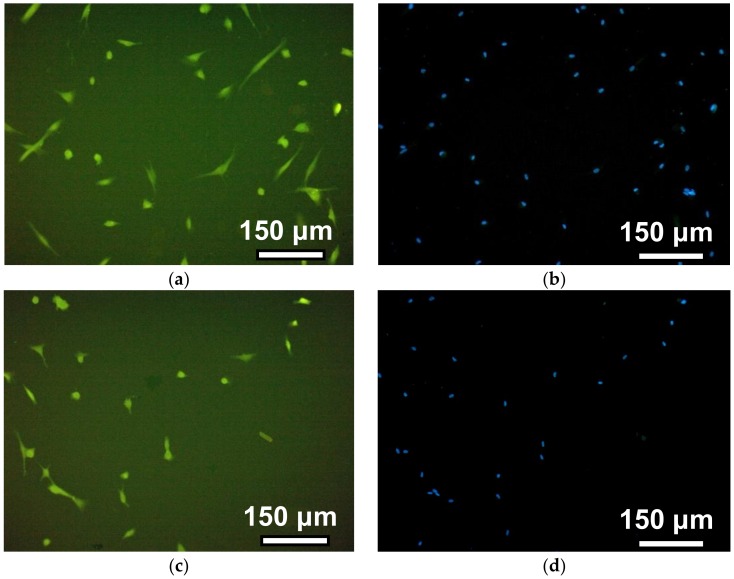

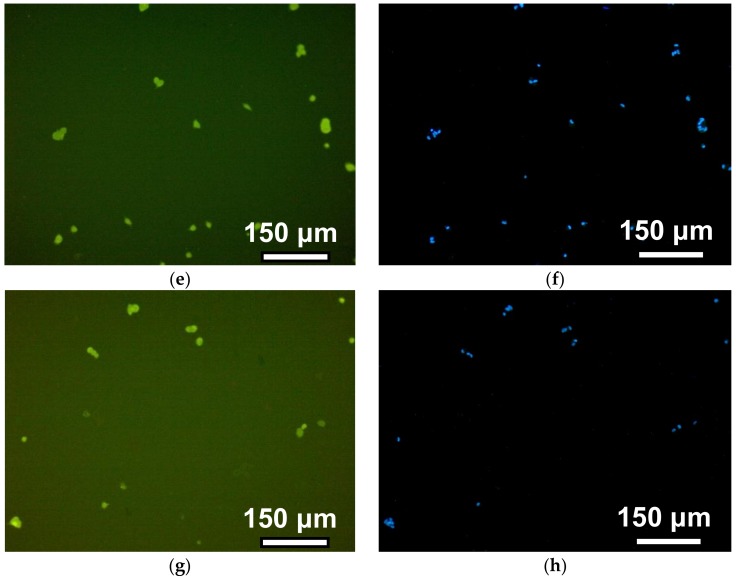
Adhesion of human dermal fibroblasts on PAAm gels with different crosslinking densities. Images were taken 12 h after seeding, staining of the cytoplasm of cells with pyrazolone yellow (**a**,**c**,**e**,**g**), and staining of nuclei with a solution of DAPI (**b**,**d**,**f**,**h**). Photos from top to bottom correspond to gels with a crosslinking density of 1:50 (**a**,**b**), 1:100 (**c**,**d**), 1:200 (**e,f**), and 1:300 (**g**,**h**).

In the first series of biological experiments, the fibroblasts’ adhesive and proliferative activities on surface of hydrogels with different cross-linking density were investigated. Figure 6 shows a series of photographs recorded in the course of the human dermal fibroblasts’ adhesion on the surface of gels with different crosslink densities (see also Table 1, Table 2 and Table 3). 

The observation time corresponds to 12 h after the cells’ disposal. The photos in the left column illustrate the morphology of the cells when the cytoplasm of fibroblasts was stained with pyrazolone yellow. The right column shows the visualization of fibroblast nuclei due to their staining with DAPI. This approach improved the cell counting accuracy in the field of view due to the significant improvement of the contrast. On the surface of PAAm-50, most fibroblasts had a typical spindle-shaped or droplet-shaped morphology with 2–3 long processes or a polygonal form with several processes of variable length. On PAAm-100, the number of such cells is noticeably smaller, whereas on gels with a crosslinking density of 1:200 and 1:300, almost all of the cells remained round. This indicates that the cells on the surface of the weakly cross-linked gels interacted with each other and formed round, dense aggregates.

**Figure 7:**
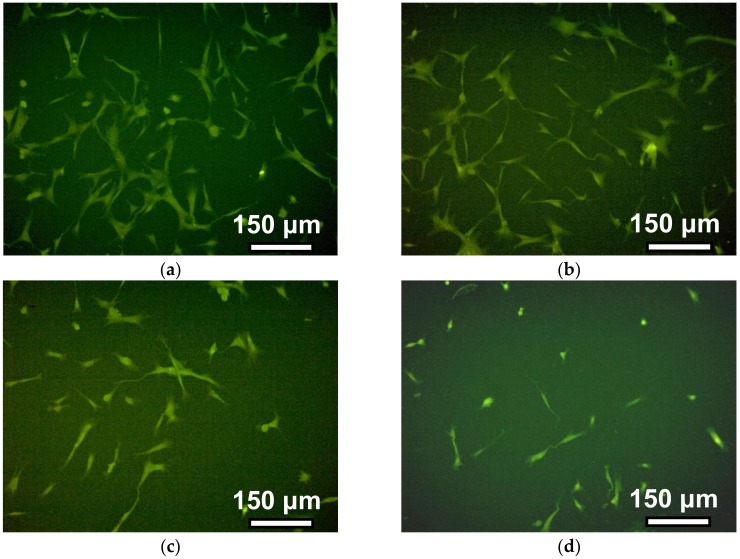
Human dermal fibroblasts on PAAm gels with different crosslinking densities after 4 days of incubation. Magnification ×100, staining of the cytoplasm of cells with pyrazolone yellow. Photos correspond to gels with a crosslinking density of 1:50 (**a**), 1:100 (**b**), 1:200 (**c**), and 1:300 (**d**).

Figure 7 shows the photograph of cells on the surface of gels with different cross-linking densities four days after cells disposal. The density of the cell monolayer decreases with the increase of the number of monomers per crosslink. In other words, the number of cells per unit of surface area in tightly cross-linked gels is greater than in weakly cross-linked gels.

Table 4 presents the results of quantitative evaluation of the adhesive and proliferative activities of fibroblasts. In general, one can see a tendency to an increase in the cells’ compatibility to gels as the crosslinking density increases, similar to the above examples (see also Figure 6; Figure 7). However, significant differences between the types of gel substrate in the number of cells in the field of view for 12 h of incubation were not established. However, the average cell area for this period was significantly different in all gels in relation to the rigidity. This indicator reflects the degree of cell spreading on the surface, and indirectly indicates the affinity of fibroblasts to it.

For four days of cells incubation, the values of the number of cells in the monolayer per the field of view were unobtrusive for PAAm-50 and PAAm-100 gels. Significant differences of this indicator were found only between these gels and the most weakly cross-linked gel (1:300). This result is expected since the degree of spreading of fibroblasts during adhesion is associated with their ability to proliferate [43,44,45]. Thus, higher adhesive and proliferative activity of fibroblasts were detected on gels with the highest density of cross-links of the polymer network, and, accordingly, with greater rigidity of the material. The obtained results are consistent with the idea that cell adhesion and proliferation depend on the elastic properties of substrates for the cultivation of biological material [17,18,19,20,43,44,45,46]. This is due to the fact that the mechanical properties of the substrate are perceived by the cytoskeleton of the cell as a type of signal regulating the morphology and proliferation of fibroblasts [44].

The adhesion level and proliferation rate of cells in control wells (culture treated polystyrene) was much higher than those on the gel surfaces (see Table 4). After 12 h incubation, the average quantity of adhered fibroblasts in one position was 42 ± 4 in control wells and 22 ± 7 on the gel with the highest cross-linking density PAA-50. Likewise, the average quantity of cells after 4 days proliferation in control and on PAA-50 was 230 ± 20 and 122 ± 35, respectively. These results confirm the fine adhesion activity and proliferation rate of primary fibroblasts, which were used in experiments. The comparable quantities of fibroblasts in control and on PAAm gels confirm the absence of cytotoxicity of gels. At the same time, the low adhesion activity of cells on the surface of PAAm gels reduce the proliferation rate of fibroblasts adhered.

In the next series of experiments, the adhesion and proliferation of fibroblasts on gels and ferrogels were tested. No magnetic field was applied during experiments except the Earth´s local magnetic field (which was not specially compensated). The results of quantitative assessment of fibroblasts are reflected in Table 5. One can see that the presence of iron oxide MNPs in the composition of the PAAm-100+Fe gel results in a significant increase in the adhesive and proliferative activities of fibroblasts. For the 12 h cultivation case, the number of human dermal fibroblasts in the field of view was on average about 1.6 times higher on the surface of the ferrogel than on the surface base polymer (blank gel). No significant differences were found in the average cell area. On the fourth day, the difference in the number of cells in the field of view between gel and ferrogel increased by about 2.5 times on average. Consequently, the cells’ compatibility to the ferrogel was significantly better than to the blank gel, on the basis of which, the magnetic composite was synthesized. Thus, the obtained results show that almost the same increment of elasticity from PAAm-100 to PAAm-50 and from PAAm-100 to PAAm-100+Fe (see Table 3) accompanies the difference in cell behavior.

Generally, the comparison of the results obtained in the two series of biological experiments is not correct. Meanwhile, based on the fact that the controls in both series are quite similar (see Table 4; Table 5), the positive effect of adding particles on the adhesion and proliferation of fibroblasts is disproportionately greater than the effect of increasing the density of crosslinking from 1:100 to 1:50. As a result, at approximately the same value of the Young modulus for the PAAm-50 gel and the PAAm-100 ferrogel, the proliferation rate of fibroblasts on the ferrogel was, on average, about two times more than on the PAAm-50. 

## 4. Discussion 

First, it is necessary to answer the fundamental question and evaluate the potential release of the magnetic nanoparticles from the composite, because at low particle concentration, cells should be mostly in contact with the polymer. Fabricated maghemite MNPs were not chemically linked to the polymeric matrix. No chemical bonding between the surface of MNPs and the subchains of gel network was provided in the synthesis. There is a possibility of Van der Waals adhesion between PAAm subchains and the surface of maghemite. This possibility was analyzed in our earlier study [43] and it was shown that the enthalpy of adhesion for PAAm chains to the surface of maghemite MNPs is negative and reached the value of –17.7 J/m^2^ in the saturated adsorption layer. This means that it is favourable for PAAm subchains to adsorb on the surface of MNPs in terms of energy. Meanwhile, adhesive interaction by itself is not sufficiently strong to immobilize MNPs in the gel network. The actual reason for the immobilization of MNPs is provided by the spatial restrictions for their translational movements. Table 1 shows that the mesh size of the network (L) for PAAm-100 was estimated as 2.2 nm. It is an order of magnitude lower than the median diameter of the PSD of maghemite MNPs. This means that MNPs are entrapped in the network of PAAm subchains and cannot escape from the network without breaking covalent chemical bonds, which comprises the gel’s molecular structure. Such special restrictions efficiently prevent the release of MNPs from ferrogel, which was never observed during its extensive washing.

The presented findings are of fundamental and application-oriented importance. From the fundamental point of view, the obtained results indicate the direct role of magnetic nanoparticles in the regulation of the processes of adhesion and proliferation of human dermal fibroblasts, independent of the rigidity of the matrix. In other words, regardless of the influence of MNPs on the elastic properties of cell matrices, the nanoparticles themselves are determinants of adhesion and proliferation of fibroblasts on the surface of a ferrogel. There are two possible mechanisms for such an impact. First, there is the direct effect of iron oxide on biological processes in cells, since we cannot exclude the contact of particles with cells on the surface of ferrogel. Literature data on this subject are very contradictory, although the results of the positive effect of ferrofluids on the morpho-functional reactions of cell cultures have been noted in a number of publications [47,48,49,50].

The second mechanism may be associated with the effect of particles on the surface features of ferrogel matrices, the degree of roughness of which significantly affects cell adhesion and proliferation [22]. Evaluation of the surface geometry of the gels is a non-trivial task due to the presence of a solvent in the composition of the ferrogel. At the same time, in the present work, an attempt was made to characterize the surface of PAA-100 gel and a ferrogel based on it with a weight fraction of 0.34% magnetic nanoparticles.

It must be mentioned that, despite special efforts of different research groups, rigorous characterization of the structure of the gels and composites of gel filled with magnetic or metallic nanoparticles is still absent in the literature and developed techniques are far from satisfactory. Some of the popular directions are freezing [51] or drying [52] of ferrogels followed by microscopy studies. There were also attempts to use atomic force microscopy to visualize the structure of a dilute system of PAAm chains and citrate-coated maghemite MNPs deposited on a mica substrate [53]. Confocal Scanning Laser Microscopy was also shown to be a useful technique [54].

**Figure 8:**
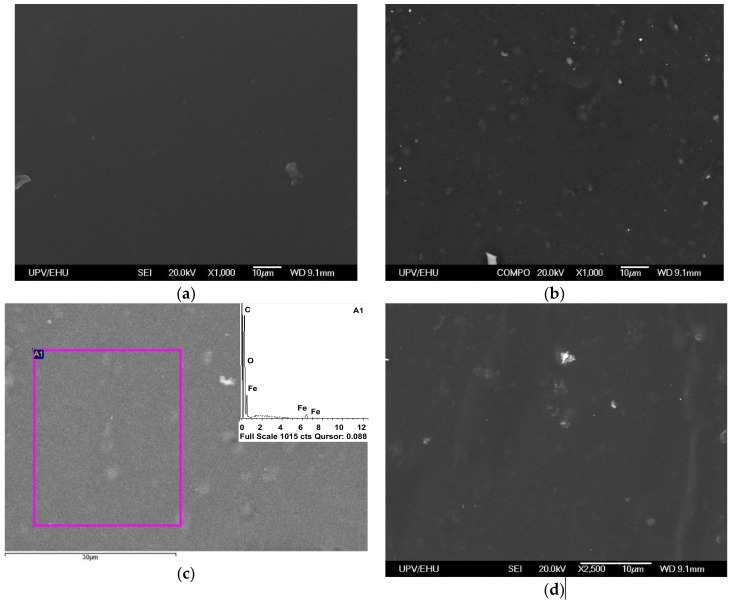
SEM studies of the surface of dried PAAm gel (**a**) and ferrogel with 0.34 wt.% γ-Fe_2_O_3_ MNPs (**b**–**d**). (**c**) Area of EDX analysis, the inset of which shows the elemental composition.

Understanding all the disadvantages of the evaluation of the structure of dried gel or ferrogel, we nevertheless take into account the widely-accepted concept that the structure of dried gel/ferrogel mirrors the structure of gel/ferrogel [55,56,57,58]. Figure 8 shows the selected results of the study of the surface peculiarities of PAAm gel and ferrogel (0.34 wt.% of iron oxide MNPs) used in biological experiments with human dermal fibroblasts. 

One can see clear a difference in the surface roughness, which is higher in the case of PAAm + γ-Fe_2_O_3_ composite (compare Table 4; Table 5). In order to insure the presence of γ-Fe_2_O_3_ MNPs in the area of observation, the elemental analysis was performed using EDX: the inset of Figure 8c clearly indicates the presence of iron in the composition. The size of observed surface peculiarities (of the order of 5 μm) is compatible with the hypothesis that they play a decisive role in the improvement of the adhesion of the human dermal fibroblasts on the surface of PAAm-100 + γ-Fe_2_O_3_ gels. The typical average size of human dermal fibroblasts is about 100 μm [59]. 

Keeping in mind that we are dealing with a dry gel surface (a one-fold reduced dimension corresponds to dry gel distances in comparison to the gel without drying), both sizes seem to be compatible. Although this argument is not strong, it is not a contradictory. As the next step, we consider using small angle neutron scattering (SANS), which has already been used to study the effect of iron oxide MNPs on the homogeneity of ferrogel structure, to show the presence of “solvent pockets” inside the PAAm ferrogel structure with the same citrate ligands [60]. 

Recently, advanced drug delivery systems based on gel-filled composites attracted special attention, while searching for enhancement of treatment efficacy of different therapeutics via the control of dose, time, or spatial delivery [61]. With this respect, gel-based scaffold biocompatibility is a crucial parameter for any kind of biomedical applications [62]. The first level of biocompatibility understanding is its estimation in the case of water based stable suspensions of the MNPs. In this respect, the LTE MNPs used in the present study serve as a very good model, as their physical and chemical properties were extensively tested in our previous studies [62,63], including hyperthermia studies and biological tests with different cell cultures [49,64,65]. Zhang et al. [55] discussed diverse mechanical force-triggered drug delivery systems and, among others, an interesting case of release by deformation of carriers under compression. Ferrogel in combination with a magnetic field sensor can be viewed as a mini robot for exact positioning of shear force activation release. 

Patient-derived cells or tissue varies dramatically from one person to another. This is a great obstacle of the development of magnetic biosensor prototypes, because one needs test samples with repeatable and stable physical properties. The present study contributes to the design of a new type of biomimetic samples for the development of magnetic biosensors [66]. Coming back to the experimental results presented above (Figure 4b), one can see that magnetization of PAAm-100 + iron oxide γ-Fe_2_O_3_ MNPs composite in the external magnetic field of 0.01T (quite comfortable for practical purposes) is about 200 A/m. A piece of ferrogel of a few mm with magnetization corresponding to a very low concentration of LTE MNPs can be detected by the magnetic field sensor operating on the basis of giant magnetoimpedance (GMI) [67,68]. The magnetoimpedance phenomenon consists of the large change of a ferromagnetic conductor under application of external magnetic field when a high frequency alternating current flows through it [68,69,70]. GMI was proposed for different biomedical applications due to its high sensitivity with respect to externally applied fields and wide variety of sensing conditions, including sensitive elements deposited onto flexible substrates [68,69]. 

**Figure 9:**
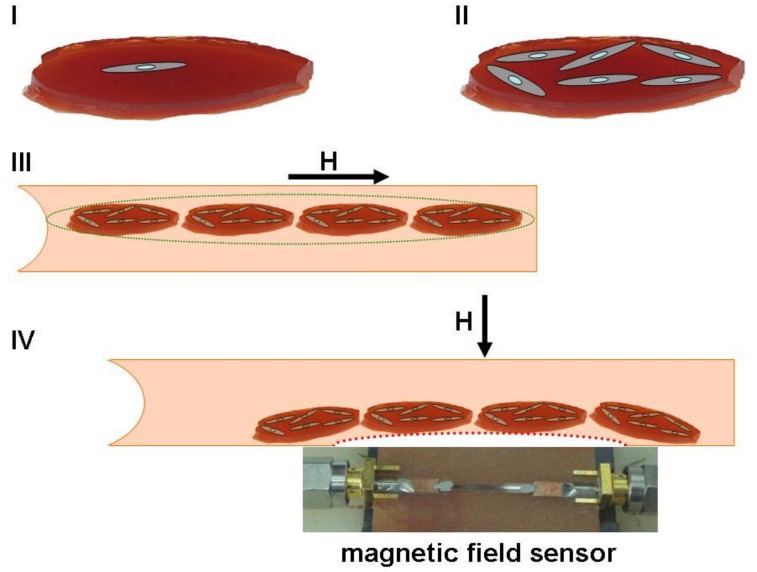
Schematic illustration of PAAm-100 + iron oxide γ-Fe_2_O_3_ MNPs-based delivery system. I—initial cell cultivation stage onto the surface of ferrogel scaffold; II—final cell cultivation stage with many adhered cells; III—intravenous delivery under applied gradient magnetic field (H) close to the direction of the blood stream velocity with dipolar chain formation formed as a result of dipole–dipole interactions between separate ferrogel scaffolds; IV—allocation of ferrogel scaffolds near the area of therapy by application of a gradient magnetic field perpendicular to the blood vessel surface. Position of the ferrogel scaffolds creating stray fields can be controlled by the magnetic field sensor. Photograph shows the GMI-based field sensitive element near the therapy area as an example.

Figure 9 shows a schematic illustration for a hypothetical regenerative medicine case with a photograph of a real GMI sensor based on a multilayered sensitive element.

Ferrogel pieces of similar shape can be used as substrates for appropriate cell cultivation and cells can be carefully counted prior to cells + ferrogel scaffold injection. Thanks to dipole–dipole interactions, separate ferrogel scaffolds will form dipolar chains under application of an appropriate magnetic field and therefore may have enhanced flexibility in combination with improved ability to move toward the area of the therapy as directed by the gradient field. Their position can be carefully controlled by an external magnetic field. The magnetic field’s configuration can be changed in the therapy area and ferrogel scaffolds can be positioned where needed and even relocated by various iterations in the feedback mode of communication with the GMI sensor and magnetic field source. Although exact applications and particular devices are still under development, from the application-oriented point of view, the obtained results show a high potential of MNPs for cellular technologies and tissue engineering [27,28,29,70]. Indeed, the addition of MNPs into gel at very low concentrations results in significant improvement of the gel-based scaffold’s compatibility with cells. 

The batch size of nanomaterials is a critically important parameter for technological and biomedical applications [26]. In our previous publications related to electrophysical techniques of fabrication of nanoparticles [25,31,33], we discussed the importance of fabrication of large batches for biomedical research. The use of the same batch of MNPs in the previous [30,31] and the present study can be considered an advantage of these works, since it allows us to exclude inter-batch variability.

## 5. Conclusions

Magnetic nanoparticles of γ-Fe_2_O_3_ were fabricated by laser target evaporation. PAAm gels with different polymer network cross-linking densities and therefore different elastic properties, as well as the polyacrylamide-based ferrogel with embedded LTE MNPs (0.34 wt.%) were synthesized. Their adhesive and proliferative potentials with respect to human dermal fibroblast cells were comparatively analyzed. It was established that at the same value of Young modulus, the adhesive and proliferative activities of the human dermal fibroblasts on the surface of ferrogel are unexpectedly much higher than that on the surface of PAAm gel. The evaluated properties of PAAm-100 + γ-Fe_2_O_3_ MNPs composites were discussed from the point of view of the possible creation of a new generation of drug delivery systems to be employed in multifunctional devices with magnetic field assisted delivery, positioning, and biosensing.

## Figures and Tables

**Table 1:** Selected parameters of the networking density for PAAm hydrogels: *α*—swelling degree; N_c_ is the number of monomer units among cross-links; L—the average distance between cross-links; L_0_—the average geometric length of the subchains.

Mark	*α* in Water	*α* in 199 Solution	N_c_	L (nm)	L_0_ (nm)	L_0_/L
PAAm-50	8.8 ± 0.1	9.0 ± 0.1	32	1.7	8.0	4.6
PAAm-100	12.6 ± 0.4	12.9 ± 0.4	52	2.2	12.9	5.8
PAAm-200	17.1 ± 0.2	17.6 ± 0.3	89	2.9	22.1	7.6
PAAm-300	21.7 ± 0.3	20.6 ± 0.4	134	3.6	33.4	9.4

**Table 2:** Tension (in N/m^2^) obtained at different deformation in the tested samples. The mean values (*n* = 4) and standard deviations are shown. The concentration of iron oxide MNPs in ferrogel was 0.34 wt.%.

Mark	Deformation (%)			
	4	8	12	16
PAAm-50	0.4 ± 0.1	1.00 ± 0.02	2.1 ± 0.1	3.5 ± 0.3
PAAm-100	0.3 ± 0.2	1.2 ± 0.2	2.0 ± 0.5	3.2 ± 0.5
PAAm-200	0.06 ± 0.01	0.18 ± 0.01	0.35 ± 0.01	0.60 ± 0.06
PAAm-300	0.07 ± 0.03	0.15 ± 0.05	0.25 ± 0.05	0.40 ± 0.06
PAAm-100 + Fe	0.56 ± 0.02	1.4 ± 0.1	2.2 ± 0.2	3.4 ± 0.4

**Table 3:** Young modulus (in kPa) obtained in the tested samples. The mean values (*n* = 4) and standard deviations are shown. The concentration of iron oxide MNPs in ferrogel was 0.34 wt.%.

PAAm-50	PAAm-100	PAAm-200	PAAm-300	PAAm-100+Fe
34 ± 6	24 ± 5	5 ± 1	3 ± 2	33 ± 4

**Table 4:** The results of the evaluation of the adhesive and proliferative activity of human fibroblasts on the surface of PAAm gels with different crosslinking density. n—the average number of adhered cells in the field of view for 12 h and four days after seeding; S—the average area (in square micrometers) of the adhered cells for 12 h after seeding; The results are presented as M´ ± m (*n* = 30). Asterisks reflect significant differences (*p* < 0.05) of the corresponding indicators with PAAm-50 and PAAm-100 gels.

	PAAm-50	PAAm-100	PAAm-200	PAAm-300	Control
**n** **(12 h** **)**	22 ± 7	20 ± 4	17 ± 3	19 ± 2	42 ± 4
**S (12 h)**	816 ± 20	683 ± 16	456 ± 9 *	430 ± 10 *	1750 ± 20
**n (4 days)**	122 ± 35	117 ± 15	81 ± 20	50 ± 10 *	230 ± 20

**Table 5:** The results of the evaluation of the adhesive and proliferative activity of human dermal fibroblasts on the surface of PAAm-100 gels with and without iron oxide γ-Fe_2_O_3_ nanoparticles. n—the average number of adhered cells in the field of view in 12 h and four days after seeding; S—the average area (in square micrometers) of the adhered cells in 12 h after seeding; The results are presented as M´ ± m (*n* = 30). Asterisks reflect significant differences (*p* < 0.05) of the corresponding indicators with PAAm-100 gels.

	PAAm-100	PAAm-100 + Fe_2_O_3_	Control
n (12 h)	20 ± 5	32 ± 7 *	36 ± 4 *
S (12 h)	775 ± 28	660 ± 20	1280 ± 20 *
n (4 days)	84 ± 11	210 ± 20 *	270 ± 20 *

## References

[1] Burdickmolly J.A., Stevence M.M. (2005). Biomaterials, artificial organs and tissue engineering. Woodhead Publishing Series Biomater..

[2] El-Sherbiny I.M., Yacoub M.H. (2013). Hydrogel scaffolds for tissue engineering: Progress and challenges. Glob. Cardiol. Sci. Pract..

[3] Smithmyer M.E., Sawickia L.A., Kloxin A.M. (2014). Hydrogel scaffolds as in vitro models to study fibroblast activation in wound healing and disease. Biomater. Sci..

[4] Morsi Y.S. (2014). Bioengineering strategies for an aortic heart valve. Int. J. Artif. Organs.

[5] Chen Y., Shiraishia N., Satokawa H., Kakugo A., Narita T., Gong J., Osada Y., Yamamoto K., Ando J. (2005). Cultivation of endothelial cells on adhesive protein-free synthetic polymer gels. Biomaterials.

[6] Wang L., Shansky J., Borselli C., Mooney D., Vandenburgh H. (2012). Design and fabrication of a biodegradable, covalently crosslinked shape-memory alginate scaffold for cell and growth factor delivery. Tissue Eng. Part A.

[7] Ansari S., Chen C., Xu X., Annabi N., Zadeh H.H., Wu B.M., Khademhosseini A., Shi S., Moshaverinia A. (2016). Muscle tissue engineering using gingival mesenchymal stem cells encapsulated in alginate hydrogels containing multiple growth factors. Ann. Biomed. Eng..

[8] Harland R., Prudhomme R. (1992). Polyelectrolyte Gels: Properties, Preparation and Applications.

[9] Pollack G. (2001). Cells, Gels and the Engines of Life.

[10] Shklyar T.F., Blyakhman F.A., Safronov A.P., Klyuzhin I.S., Pollack G. (2008). A correlation between mechanical and electrical properties of the synthetic hydrogel chosen as an experimental model of cytoskeleton. Biophysics.

[11] De Rossi D., Kajiwara K., Osada Y., Yamauchi A. (1991). Polymer Gels: Fundamentals and Biomedical Applications.

[12] Hirotsu S. (1993). Responsive Gels: Volume Transitions II, Advances in Polymer Sciences.

[13] Safronov A.P., Shakhnovich M.A., Kalganov A., Shklyar T.F., Blyakhman F.A., Pollack G.H. (2011). DC electric fields produce periodic bending of polyelectrolyte gels in dc electric field. Polymer.

[14] Blyakhman F.A., Safronov A.P., Zubarev A.Yu., Shklyar T.F., Dinislamova O.A., Lopez-Lopez M.T. (2016). Mechanoelectrical transduction in the hydrogel-based biomimetic sensors. Sens. Actuators A.

[15] Kandow C., Georges P., Janmey P., Beningo K. (2007). Polyacrylamide hydrogels for cell mechanics: steps toward optimization and alternative uses. Methods Cell Biol..

[16] Vignaud T., Ennomani H. (2014). Polyacrylamide hydrogel micropatterning. Methods Cell Biol.

[17] Dobreikina A., Shklyar T., Safronov A., Blyakhman F. (2018). Biomimetic gels with chemical and physical interpenetrating networks. Polym. Int..

[18] Sun M., Chi G., Li P., Lv S., Xu J., Xu Z., Xia Y., Tan Y., Xu J., Li L. (2018). Effects of matrix stiffness on the morphology, adhesion, proliferation and osteogenic differentiation of mesenchymal stem cells. Int. J. Med. Sci..

[19] Discher A., Janmey P., Wang Y. (2005). Tissue cells feel and respond to the stiffness of their substrate. Science.

[20] Yeung T., Georges P.C., Flanagan L.A., Marg B., Ortiz M., Funaki M., Zahir N., Ming W., Weaver V., Janmey P.A. (2005). Effects substrate stiffness on cell morphology, cytoskeletal structure, an adhesion. Cell Motil. Cytoskeleton.

[21] Cretu A., Castagnino P., Assoian R. (2010). Studying the effects of matrix stiffness on cellular function using acrylamide-based hydrogels. J. Vis. Exp..

[22] Trappmann A., Gautrot J., Connelly J., Strange D., Li Y., Oyen M., Cohen Stuart M., Boehm H., Li B., Vogel V. (2012). Extracellular-matrix tethering regulates stem-cell fate. Nat. Mater..

[23] Li Y., Huang G., Zhang X., Li B., Chen Y., Lu T., Lu T.J., Xu F. (2013). Magnetic hydrogels and their potential biomedical applications. Adv. Funct. Matters.

[24] Kennedy S., Roco C., Délérisa A., Spoerria P., Cezara C., Weavera J., Vandenburghd H., Mooney D. (2018). Improved magnetic regulation of delivery profiles from ferrogels. Biomaterials.

[25] Blyakhman F.A., Buznikov N.A., Sklyar T.F., Safronov A.P., Golubeva E.V., Svalov A.V., Sokolov S.Yu., Melnikov G.Yu., Orue I., Kurlyandskaya G.V. (2018). Mechanical, Electrical and magnetic properties of ferrogels with embedded iron oxide nanoparticles obtained by laser target evaporation: focus on multifunctional biosensor applications. Sensors.

[26] Grossman J.H., McNeil S.E. (2012). Nanotechnology in Cancer Medicine. Phys. Today.

[27] Hou R., Zhang G., Du G., Zhan D., Cong Y., Cheng Y., Fu J. (2013). Magnetic nanohydroxyapatite/PVA composite hydrogels for promoted osteoblast adhesion and proliferation. Colloids Surf. B.

[28] Van Berkum S., Dee J., Philipse P., Erne B. (2013). Frequency-dependent magnetic susceptibility of magnetite and cobalt ferrite nanoparticles embedded in PAA hydrogel. Int. J. Mol. Sci..

[29] Lopez-Lopez M.T., Scionti G., Oliveira A.C., Duran J.D.G., Campos A., Alaminos M. (2015). Generation and characterization of novel magnetic field-responsive biomaterials. PLoS ONE.

[30] Blyakhman F.A., Safronov A.P., Zubarev A.Yu., Shklyar T.F., Makeyev O.G., Makarova E.B., Melekhin V.V., Larrañaga A., Kurlyandskaya G.V. (2017). Polyacrylamide ferrogels with embedded maghemite nanoparticles for biomedical engineering. Results Phys..

[31] Blyakhman F.A., Safronov A.P., Makeyev O.G., Melekhin V.V., Shklyar T.F., Zubarev A.Yu., Makarova E.B., Sichkar D.A., Rusinova M.A., Sokolov S.Y., Kurlyandskaya G.V. (2018). Effect of the polyacrylamide ferrogel elasticity on the cell adhesiveness to magnetic composite. J. Mech. Med. Biol..

[32] Coey J.M.D. (2010). Magnetism and Magnetic Materials.

[33] Safronov A.P., Beketov I.V., Komogortsev S.V., Kurlyandskaya G.V., Medvedev A.I., Leiman D.V., Larranaga A., Bhagat S.M. (2013). Spherical magnetic nanoparticles fabricated by laser target evaporation. AIP Adv..

[34] Zhang Y., Chen Y., Westerhoff P., Hristovski K., Crittenden J.C. (2008). Stability of commercial metal oxide nanoparticles in water. Water Res..

[35] Terzian T.V., Shcherbinin S.V., Beketov I.V., Fernandez Armas S., Marcano Prieto L., Safronov A.P., Andrey V., Svalov A.V., Kurlyandskaya G.V. (2017). Scanning electron microscopy for structural evaluation of metallic nanoparticles/polymer composites designed for high frequency applications. Materials, Methods & Technologies ISSN 1314-7269. J. Int. Sci. Publications.

[36] Quesada-Perez M., Maroto-Centeno J.A., Forcada J., Hidalgo-Alvarez R. (2011). Gel swelling theories: the classical formalism and recent approaches. Soft Matter.

[37] Katchalsky A., Lifson S., Exsenberg H. (1951). Equation of swelling for polyelectrolyte gels. J. Polym. Sci..

[38] Rubinstein M., Colby R.H. (2003). Polymer Physics.

[39] Benguigui L., Boué F. (1999). Homogeneous and inhomogenous polyacrylamide gels as observed by small angle neutron scattering: A connection with elastic properties. Eur. Phys. J. B.

[40] Engler A.J., Sen S., Sweeney H.L., Discher D.E. (2006). Matrix elasticity directs stem cell lineage specification. Cell.

[41] Galicia J.A., Sandre O., Cousin F., Guemghar D., Menager C., Cabuil V. (2003). Designing magnetic composite materials using aqueous magnetic fluids. J. Phys. Cond. Mat..

[42] Galbraith C.G., Sheetz M.P. (1998). Forces on adhesive contacts affect cell function. Cell Biol..

[43] Mih J.D., Marinkovic A., Liu F., Sharif A.S., Tschumperlin D.J. (2012). Matrix stiffness reverses the effect of actomyosin tension on cell proliferation. J. Cell Sci..

[44] Mullen C.A., Vaughan T.J., Voisin M.C., Mc Namara L.M. (2014). Cell morphology and focal adhesion location alters internal cell stress. J. R. Soc. Interface.

[45] Safronov A.P., Samatov O.M., Tyukova I.S., Mikhnevich E.A., Beketov I.V. (2016). Heating of polyacrylamide ferrogel by alternating magnetic field. J. Magn. Magn. Mat..

[46] Pareta R., Taylor E., Webster T. (2008). Increased osteoblast density in the presence of novel calcium phosphate coated magnetic nanoparticles. Nanotechnology.

[47] Yang J.-X., Tang W.-L., Wang X.-X., Yang, (2010). Superparamagnetic iron oxide nanoparticles may affect endothelial progenitor cell migration ability and adhesion capacity. Cytotherapy.

[48] Sun J., Wang S., Zhao D., Hun F.H., Weng L., Liu H. (2011). Cytotoxicity, permeability, and inflammation of metal oxide nanoparticles in human cardiac microvascular endothelial cells. Cell Biol. Toxicol..

[49] Denisova T.P., Simonova E.V., Kokorina L.A., Maximova E.N., Samatov O.M., Safronov A.P., Kurlyandskaya G.V. (2018). Heterogeneity of population of microorganisms grown in presence of iron oxide maghemite nanoparticles. EPJ Web Conf..

[50] Moscoso-Londoño O., Gonzalez J.S., Muraca D., Hoppe C.E., Alvarez V.A., López-Quintela A., Socolovsky L.M., Pirota K.R. (2013). Structural and magnetic behavior of ferrogels obtained by freezing thawing of polyvinyl alcohol/poly(acrylic acid) (PAA)-coated iron oxide nanoparticles. Eur. Polym. J..

[51] Helminger M., Wu B., Kollmann T., Benke D., Schwahn D., Pipich V., Faivre D., Zahn D., Cölfen H. (2014). Synthesis and characterization of gelatin-based magnetic hydrogels. Adv. Funct. Mater..

[52] Galicia J.A., Cousin F., Dubois E., Sandre O., Cabuil V., Perzynski R. (2011). Local structure of polymeric ferrogels. J. Magn. Magn. Mater..

[53] Van der Zande B.M.I., Page`s L., Hikmet R.A.M., van Blaaderen A. (1999). Optical properties of aligned rod-shaped gold particles dispersed in poly(vinyl alcohol) films. J. Phys. Chem. B.

[54] Zhang Y., Yu J., Bomba H.N., Zhu Y., Gu Z. (2016). Mechanical force-triggered drug delivery. Chem. Rev..

[55] Shin B.Y., Cha B.G., Jeong J.H., Kim J. (2017). Injectable macroporous ferrogel microbeads with a high structural stability for magnetically actuated drug delivery. ACS Appl. Mater. Interfaces.

[56] Shankar A., Safronov A.P., Mikhnevich E.A., Beketov I.V., Kurlyandskaya G.V. (2017). Ferrogels based on entrapped metallic iron nanoparticles in a polyacrylamide network: extended Derjaguin–Landau–Verwey–Overbeek consideration, interfacial interactions and magneto deformation. Soft Matter.

[57] Liu T.-Y., Chan T.-Y., Wang K.-S., Tsou H.-M. (2015). Influence of magnetic nanoparticle arrangement in ferrogels for tunable biomolecule diffusion. RSC Adv..

[58] Gabbott C., Sun T. (2017). Mechanistic insights of cells in porous scaffolds via integrated culture technologies. J. Life Sciences.

[59] Galicia J.A., Cousin F., Dubois E., Sandre O., Cabuila V., Perzynski R. (2009). Static and dynamic structural probing of swollen polyacrylamide ferrogels. Soft Matt..

[60] Roca A.G., Costo R., Rebolledo A.F., Veintemillas-Verdaguer S., Tartaj P., González-Carreño T., Morales M.P., Serna C.J. (2009). Progress in the preparation of magnetic nanoparticles for applications in biomedicine. J. Phys. D Appl. Phys..

[61] Novoselova I.P., Safronov A.P., Samatov O.M., Beketov I.V., Medvedev A.I., Kurlyandskaya G.V. (2016). Water based suspensions of iron oxide obtained by laser target evaporation for biomedical applications. J. Magn. Magn. Mater..

[62] Kurlyandskaya G.V., Novoselova I.P., Schupletsova V.V., Andrade R., Dunec N.A., Litvinova L.S., Safronov A.P., Yurova K.A., Kulesh N.A., Dzyuman A.N., Khlusov I.A. (2017). Nanoparticles for magnetic biosensing systems. J. Magn. Magn. Mater..

[63] Blanc-Béguin F., Nabily S., Gieraltowski J., Turzo A., Querellou S., Salaun P.Y. (2009). Cytotoxicity and GMI bio-sensor detection of maghemite nanoparticles internalized into cells. J. Magn. Magn. Mater..

[64] Coïsson M., Barrera G., Appino C., Celegato F., Martino L., Safronov A.P., Kurlyandskaya G.V., Tiberto P. (2019). Specific loss power measurements by calorimetric and thermal methods on γ-Fe2O3 nanoparticles for magnetic hyperthermia. J. Magn. Magn. Mater..

[65] Buznikov N.A., Safronov A.P., Orue I., Golubeva E.V., Lepalovskij V.N., Svalov A.V., Chlenova A.A., Kurlyandskaya G.V. (2018). Modelling of magnetoimpedance response of thin film sensitive element in the presence of ferrogel: Next step toward development of biosensor for in tissue embedded magnetic nanoparticles detection. Bios. Bioelectr..

[66] Beach R., Berkowitz A. (1994). Sensitive field-and frequency-dependent impedance spectra of amorphous FeCoSiB wire and ribbon. J. Appl. Phys..

[67] Antonov A.S., Gadetskii S.N., Granovskii A.B., D’yachkov A.L., Paramonov V.P., Perov N.S., Prokoshin A.F., Usov N.A., Lagar’kov A.N. (1997). Giant magnetoimpedance in amorphous and nanocrystalline multilayers. Phys. Met. Metallogr..

[68] Uchiyama T., Mohri K., Honkura Y., Panina L.V. (2012). Recent advances of pico-Tesla resolution magneto-impedance sensor based on amorphous wire CMOS IC MI Sensor. IEEE Trans. Magn..

[69] Kurlyandskaya G.V., Fernández E., Svalov A., Burgoa Beitia A., García-Arribas A., Larrañaga A. (2016). Flexible thin film magnetoimpedance sensors. J. Magn. Magn. Mater..

[70] Díaz E., Valle M.B., Ribeiro S., Lanceros-Mendez S., Barandiarán J.M. (2018). Development of magnetically active scaffolds for bone regeneration. Nanomaterials.

